# Backfilling Patellar and Tibial Harvest Sites Using Autologous Bone Reamings and Anterior Cruciate Ligament Remnant Tissue During Bone–Patellar Tendon–Bone Anterior Cruciate Ligament Reconstruction

**DOI:** 10.1002/atn2.70318

**Published:** 2026-10-21

**Authors:** Jack H. Kramer, Donovan A. Bronstein, Olivia M. Jochl, Eddie K. Afetse, Daniel R. G. Lind, Joseph J. Ruzbarsky

**Affiliations:** ^1^ University of Illinois College of Medicine Rockford Illinois U.S.A.; ^2^ The Steadman Clinic Vail Colorado U.S.A.; ^3^ Hospital for Special Surgery New York New York U.S.A.; ^4^ Wake Forest School of Medicine Winston Salem North Carolina U.S.A.

## Abstract

The bone–patellar tendon–bone autograft remains a common and often first‐choice option for anterior cruciate ligament reconstruction. Nevertheless, a subset of patients suffer from chronic anterior knee pain at the site of graft harvest. Although prior literature has indicated that backfilling of the patellar and tibial defects decreases the risk of donor site pain, sourcing and harvesting of the graft material, as well as the management of the paratenon and underlying tendon defect, remains variable. This technical note and associated video describe our preferred technique for the backfilling of these defects using both bone and soft‐tissue grafts, with paratenon preservation to improve containment and potentially incorporation of grafted material.

## Graphical abstract

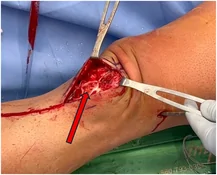

**VIDEO 1:** The video shows our preferred technique of a bone–patella–bone harvest with preservation of the paratenon for later closure. After preservation of the paratenon, a standard bone–patella–bone autograft is harvested and prepared for passage on the back table. During preparation, the bone trimmings are collected for later backfilling of the bone block harvest sites. Next, the ruptured anterior cruciate ligament (ACL) remnants are debrided and collected using a 5.5‐mm shaver and a Tobra Medical Bone Basket (Raleigh, NC). After complete resection of the ruptured ACL remnant, the Tobra Medical Bone Basket is opened, and the soft tissue collected is dried for later reimplantation. Next, the same or an additional Tobra Medical Bone Basket is used to collect bone reamings from both the femoral socket and the tibial tunnel creation. After ACL graft passage and fixation, a No. 0 Vicryl suture is placed into the superior portion of the harvested patella tendon, and the previously collected bony graft trimmings, followed by bone reamings, are packed into the patella and tibial harvest sites. The previously collected ACL remnants are then packed into the patellar tendon void anterior to Hoffa's fat pad. Finally, the paratenon is closed in a watertight fashion over top using a running No. 0 Vicryl suture, followed by standard layered closure. Video content can be viewed at https://doi.org/10.1002/atn2.70318.

The bone–patellar tendon–bone (BPTB) autograft remains the gold standard of anterior cruciate ligament (ACL) reconstruction, particularly in athletes and young patients.^1^ The BPTB autograft allows for bone‐to‐bone healing and high rates of return to sport and leaves the hamstrings intact for active patients. Nevertheless, harvesting of the BPTB autograft inherently leads to patellar and tibial defects that can increase the odds of anterior knee pain.^2^, ^3^ Several studies have shown that backfilling the defects with bone after the reconstruction decreases the risk of anterior knee pain and can restore normal biomechanical strain and forces.^4^, ^5^, ^6^, ^7^ Although filling these defects with bone graft has been shown to decrease anterior knee pain, sourcing and harvesting the graft material remains variable and challenging.^4^

As a result, this technical note and associated video (Video 1) describe our preferred technique for autograft bone and soft‐tissue collection during ACL reconstruction with the BPTB autograft. In addition to harvesting bone grafts from the femoral and tibial tunnels, we arthroscopically collect soft‐tissue autograft from the ruptured native ACL stump to serve as a collagen fill and scaffold for the tendon portion of the graft harvest site. We also emphasize the preservation of the paratenon and its closure at the end of the procedure to contain the graft material and promote tendon regeneration.

## SURGICAL TECHNIQUE

### Indications and Preoperative Planning

To assess the ACL tear, the suitability of the patella tendon, and potential concomitant pathologies in the joint, patients undergo preoperative imaging that includes anteroposterior, Rosenberg, lateral, Merchant, and standing alignment radiographs, as well as magnetic resonance imaging.

### Preoperative Anesthesia

General anesthesia is administered. Regional anesthesia includes adductor and infiltration posterior to artery and capsule of knee nerve blocks.

### Patient Positioning, Skin Prep, and Surgical Draping

After induction of general anesthesia, the patient is placed supine on a standard operating room bed and draped in the standard fashion. A lateral thigh post is placed on the operative side. Postpositioning aids in applying valgus pressure for the visualization of the medial compartment during arthroscopy. A sterile tourniquet is inflated to 200 to 250 mmHg after the limb is exsanguinated. Distal to the tourniquet, a 1000 drape is placed before beginning chlorhexidine skin preparation and standard extremity draping. An impervious stockinette is then placed over the lower leg and foot.

### BPTB Autograft Harvest

First, a 9‐cm paramedial, longitudinal incision along the ipsilateral knee is performed from the inferior pole of the patella to 2 cm distal to the tibial tubercle. After incising the skin and subcutaneous tissue, the paratenon is carefully dissected and preserved for later watertight closure. The central third of the tendon is identified, and the borders are marked. Then, a rectangular bone block, approximately 22 mm × 9.5 to 10 mm, is obtained from the inferior pole of the patella using an oscillating saw and osteotome. The central third of the patellar tendon is then sharply released from the underlying fat pad and harvested to the tibial tubercle, where a second bone block, measuring approximately 25 mm in length and 9.5 to 10 mm wide, is removed in the same fashion. On a separate table, the graft is bulletized before suturing both bone blocks for passage. Excessive autograft bone from this step is collected for later bone grafting. The graft is then submerged until use in 500 mL of normal saline combined with 1 g of Vancomycin. The harvest site is left open at this stage because bone and tissue reamings will be used later to backfill the bony defects and close the tendon gap (Figure 1). The standard arthroscopic anteromedial and anterolateral portals are created within this longitudinal incision.

**FIGURE 1:**
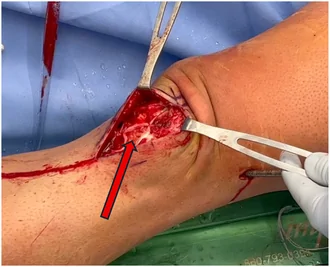
Harvest site defect of the anterior knee prior to bone or soft‐tissue grafting after taking the bone from the inferior pole of the patella and the tibial tubercle.

### ACL Reconstruction With Tissue Debridement and Bone Collection

Before beginning the arthroscopic portion of the surgery, the 4.5‐ or 5.5‐mm shaver is fitted with the Tobra Medical Bone Basket (Raleigh, North Carolina) (Figure 2). Using a large‐bore shaver can better facilitate graft collection compared with a small, low‐profile shaver (Table 1). Approaching the lateral femoral notch and the tibial intercondylar eminence through standard anteromedial and anterolateral portals, the remnant ACL stumps are debrided and collected. The tissue collector is then removed from the shaver, and the soft‐tissue remnants are collected to dry in a specimen cup. A tibial tunnel is created to exit at the native ACL footprint with an origin on the anteromedial tibia. A femoral socket is drilled through the anatomic origin of the ACL on the lateral femoral condyle using an outside‐in drilling technique. Bone reamings from both tunnels are again collected using the Tobra Medical Bone Basket (Figure 2). The previously prepared graft is then passed in a retrograde fashion using a shuttling suture through the tibial tunnel and docked into the femoral tunnel with its bone blocks flush against the tunnel walls. The femoral bone plug is then fixated using a 7 mm × 25 mm titanium interference screw made by Smith & Nephew (Andover, MA) (Figure 3) parallel to the bone plug. Finally, after tensioning the graft while cycling the knee for 20 cycles, the femoral bone block is fixed with the knee in 20° of flexion with a posterior drawer force using a titanium interference screw.

**FIGURE 2:**
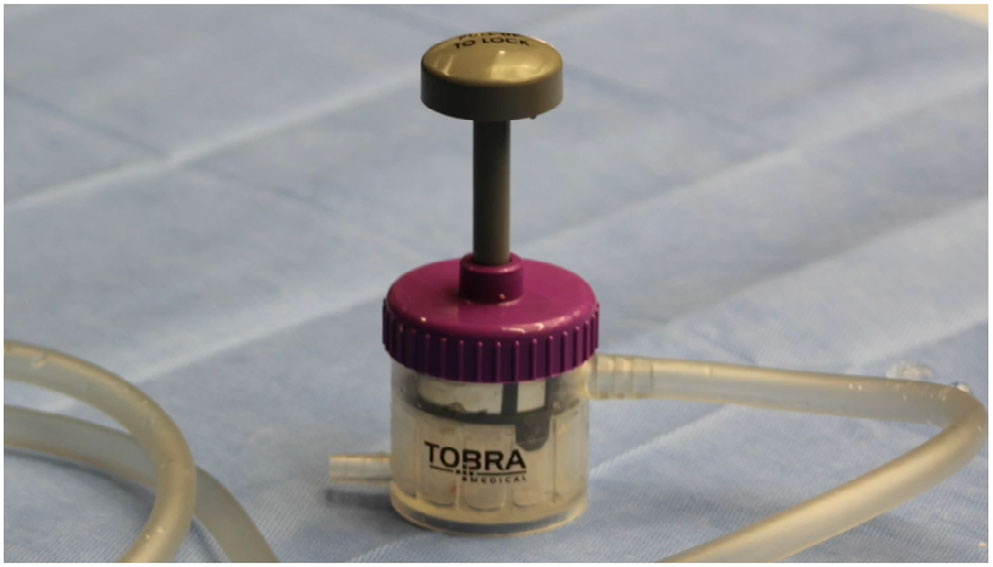
Tobra Medical Bone Basket (Raleigh, NC), which is used to collect the bone reamings from the large‐bore shaver to be used later for backfilling the defect.

**TABLE 1:** Advantages and Disadvantages of Bone and Soft‐Tissue Grafting in Bone–Patellar Tendon–Bone ACL Reconstruction

**Advantages**	**Disadvantages**
May decrease anterior knee pain post‐ACL reconstruction	High cost of arthroscopic bone collection tools
Aesthetically results in a normal anatomic appearance rather than “sunken in”	Increased operative time for graft collection
May decrease the risk of fracture or patellar tendon injury or tendonitis	Increased time for closure

ACL, anterior cruciate ligament.

**FIGURE 3:**
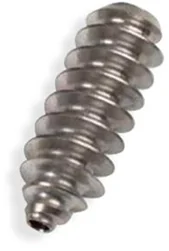
Seven‐by‐twenty‐five‐millimeter Smith & Nephew Titanium Interference Screw (Andover, MA) that was used to fix the femoral bone plug after passing the prepared graft through the tibial tunnel to the femoral tunnel, with the bone blocks flush against the walls.

### Backfilling of Harvest Sites

After ACL reconstruction, meticulous hemostasis is achieved intra‐articularly and extra‐articularly. A single No. 0 Vicryl suture is placed at the base of the patella–bone block through the proximal patella tendon adjacent to the patella to create a pocket for capturing the bone graft. By suturing at the base of the patella tendon, this technique avoids shortening it and risking iatrogenic injury (Table 1). This process is not repeated for the tibial bone harvesting site because the area is well contained and rectangular, allowing for easier containment of the backfilling bone graft. Next, the remnant bone from the graft preparation and tunnel drilling is used to first fill the patella–bone block harvest site, followed by the tibia bone block harvest site (Figure 4). The reamings from the tibial and femoral ACL reconstruction tunnels, collected from the Tobra Medical Bone Basket, are spread evenly to the patella and tibia harvest site defects. Next, the previously collected soft‐tissue remnants from the debrided ACL stump are packed directly into the patellar tendon void anterior to the underlying fat pad. To facilitate containment of the bone remnants and to aid in healing, the paratenon is closed overtop using a running, locked No. 0 Vicryl suture (Figure 5). The paratenon closure begins superiorly with the knee in full extension and is finished with the knee at 30° of flexion. The knee is cycled through a full range of motion before closure in layers.

**FIGURE 4:**
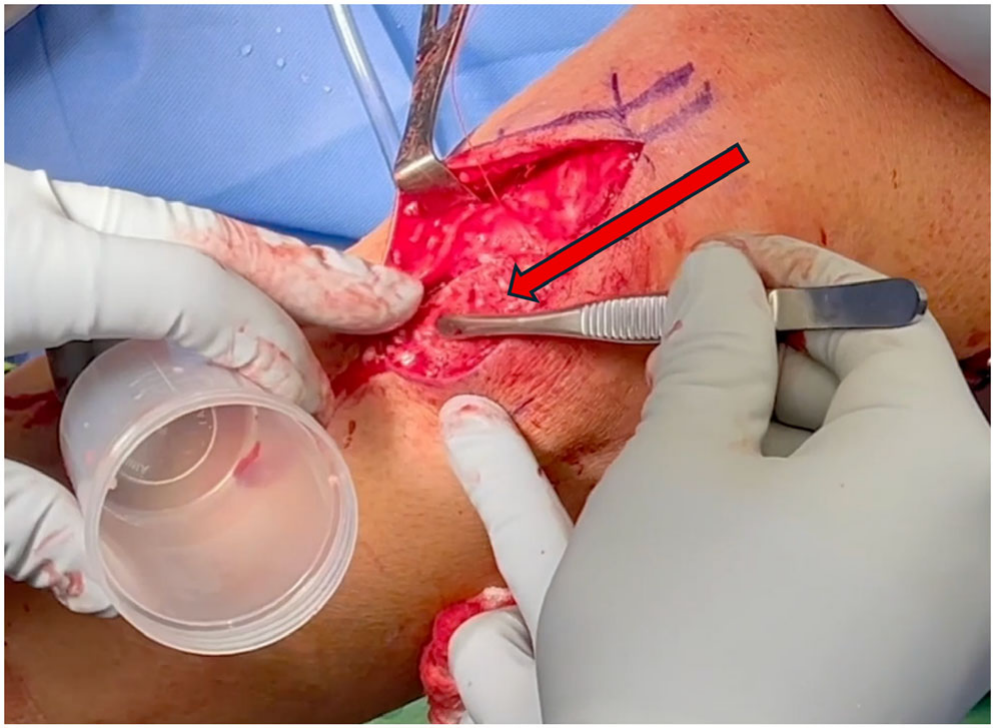
Backfilling of tibial harvest site on the anterior knee with collected autograft bone from the Tobra Medical Bone Basket.

**FIGURE 5:**
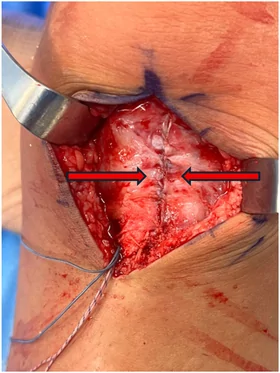
Overtop closed paratenon using a running, locked No. 0 Vicryl suture to facilitate containment of the bone remnants and to aid in healing. The paratenon closure begins superiorly with the knee in full extension and is finished with the knee at 30° of flexion.

### Rehabilitation

For 2 weeks postoperatively, the patient wears a T‐SCOPE brace locked in extension with ambulation but is allowed full weightbearing as well as full range of motion for isolated ACL reconstructions. In the setting of meniscal repairs or other concomitant procedures, weightbearing may be delayed 2 or more weeks, and range of motion may be limited to 0° to 90°. Depending on the cutting and pivoting demands, return to sport is expected between 9 and 12 months.

## DISCUSSION

Anterior knee pain is a common complication after ACL reconstruction with the BPTB autograft. Although bone grafting of the patella and tibial harvest sites has been shown to reduce anterior knee pain, obtaining sufficient autograft material remains challenging.^5^, ^8^, ^9^ Typically, only bone is collected and used for backfilling.^10^ One randomized controlled trial also showed that using only bone did not reduce anterior knee pain.^11^ The present technique describes the additional collection of soft tissue from the ruptured ACL stump to further backfill the graft defect. Our described technique also emphasizes preserving the paratenon to capture all the grafting and facilitate healing.^12^

Based on the authors’ clinical experience, ACL grafting with bone and soft tissue as described here offers a viable approach for ACL reconstructions with patella and tibia harvest site defects. This technique may reduce anterior knee pain postoperatively, keeps an aesthetically normal anatomic appearance of the patella tendon harvest site rather than a “sunken in” one, and may decrease the risk of fracture, future patella tendon injury, or tendonitis. Potential disadvantages of this technique include the cost of arthroscopic bone collection tools and an increased surgical and anesthesia time needed for graft collection and site closure (Table 2). These consequences as well as the patient's goals and background should be considered when using the described technique.

**TABLE 2:** Pearls and Pitfalls

**Pearls**	**Pitfalls**
Use of a large‐bore shaver can better facilitate graft collection compared with a small, low‐profile shaver	Always examine the knee for additional bone shavings because these can collect posterolaterally or posteromedially
Drying of the ACL stump soft‐tissue remnants will facilitate later handling for grafting	When contouring the graft, collect all excess bone and use these pieces first when backfilling the bone block defects prior to adding the morselized bone from the tunnels
Use a single No. 0 Vicryl suture at the base of the patellar tendon to avoid shortening it and risking iatrogenic patella baja	Take time with the paratenon dissection distally where the tissue thins

ACL, anterior cruciate ligament.

## DISCLOSURES

The author (J.J.R.) declares the following financial interests/personal relationships which may be considered as potential competing interests: J.J.R. receives personal fees from Smith & Nephew and Tobra Medical for consulting. The other authors (J.H.K., D.A.B., O.M.J., E.K.A., D.R.G.L.) declare that they have no known competing financial interests or personal relationships that could have appeared to influence the work reported in this paper.
